# Mechanically-Loaded Breast Cancer Cells Modify Osteocyte Mechanosensitivity by Secreting Factors That Increase Osteocyte Dendrite Formation and Downstream Resorption

**DOI:** 10.3389/fendo.2018.00352

**Published:** 2018-07-03

**Authors:** Wenbo Wang, Blayne A. Sarazin, Gabriel Kornilowicz, Maureen E. Lynch

**Affiliations:** ^1^Department of Mechanical and Industrial Engineering, University of Massachusetts, Amherst, MA, United States; ^2^Department of Mechanical Engineering, University of Colorado, Boulder, CO, United States

**Keywords:** osteocyte, mechanical loading, fluid flow, bone, breast cancer, mechanobiology

## Abstract

Advanced breast cancer predominantly metastasizes to the skeleton, at which point patient prognosis significantly declines concomitant with bone loss, pain, and heightened fracture risk. Given the skeleton's sensitivity to mechanical signals, increased mechanical loading is well-documented to increase bone mass, and it also inhibited bone metastatic tumor formation and progression *in vivo*, though the underlying mechanisms remain under investigation. Here, we focus on the role of the osteocyte because it is the primary skeletal mechanosensor and in turn directs the remodeling balance between formation and resoprtion. In particular, osteocytic dendrites are important for mechanosensing, but how this function is altered during bone metastatic breast cancer is unknown. To examine how breast cancer cells modulate dendrite formation and function, we exposed osteocytes (MLO-Y4) to medium conditioned by breast cancer cells (MDA-MB231) and to applied fluid flow (2 h per day for 3 days, shear stress 1.1 Pa). When loading was applied to MLOs, dendrite formation increased despite the presence of tumor-derived factors while overall MLO cell number was reduced. We then exposed MLOs to fluid flow as well as media conditioned by MDAs that had been similarly loaded. When nonloaded MLOs were treated with conditioned media from loaded MDAs, their dendrite formation increased in a manner similar to that observed due to loading alone. When MLOs simultaneously underwent loading and treatment with loaded conditioned media, dendrite formation was greatest. To understand potential molecular mechanisms, we then investigated expression of genes related to osteocyte maturation and dendrite formation (E11) and remodeling (RANKL, OPG) as well as osteocyte apoptosis. E11 expression increased with loading, consistent with increased dendrite formation. Though loaded conditioned media decreased MLO cell number, apoptosis was not detected via TUNEL staining, suggesting an inhibition of growth instead. OPG expression was inhibited while RANKL expression was unaffected, leading to an overall increase in the RANKL/OPG ratio with conditioned media from loaded breast cancer cells. Taken together, our results suggest that skeletal mechanical loading stimulates breast cancer cells to alter osteocyte mechanosensing by increasing dendrite formation and downstream resorption.

## Introduction

The skeleton is the most common site for breast cancer metastasis, where roughly 3 in 4 patients with advanced breast cancer develop incurable bone metastases (1). Once bone metastasis occurs, the lesions are overwhelmingly osteolytic, causing significant declines in prognosis (2) and severe skeletal complications such as bone pain and fracture (3). Once in bone, metastatic tumor cells dysregulate the normal bone remodeling process and initiate bone destruction to release vital growth factors from the bone matrix that literally “feed” the tumor cells (4). Thus, tumor growth and osteolysis are closely correlated. Currently, therapeutic strategies that target this relationship include both anti-osteoclastic and anti-tumorigenic effects [e.g., bisphosphonates (5, 6), denosumab (7)], and many new, more targeted approaches are being developed [e.g., nanomedicine that combines bisphosphonates with chemotherapies (8, 9)]. Despite these advances, most treatment options do not recover bone that has been lost with osteolysis, and improving the ability of these strategies to inhibit tumorigenesis and osteolysis long-term remains a goal.

Dynamic mechanical loading has recently been established as an important microenvironmental factor in bone metastatic cancer. In healthy bone, increased loading is well-documented to shift the remodeling balance toward bone formation by upregulating bone-forming osteoblasts and inhibiting bone-resorbing osteoclasts (10). Because this effect is the opposite of how bone metastatic tumors cells shift remodeling, loading is under investigation as a potential method of opposing cancer-induced bone disease. Supporting this hypothesis, increased mechanical loading in mouse models protected against bone metastatic breast cancer-induced osteolysis while increasing bone formation (11) and it inhibited multiple myeloma bone metastasis (12). In a 3D model of bone metastasis, applied mechanical loading inhibited breast cancer cell expression of genes interfering with remodeling (11). Furthermore, mechanical loading modulated interactions between breast cancer cells and mesenchymal stem cells (13), which are the progenitor cells that give rise to osteoblasts as well as to osteocytes, a specialized mechanosensory bone cell whose role in cancer-induced bone disease has only recently been recognized.

Osteocytes are the most abundant cell type in the skeleton, forming a complex, interconnected network throughout the skeleton. They are critical for sensing mechanical and chemical signals from all over the skeleton, and they integrate these signals to appropriately coordinate the downstream activities of osteoblasts and osteoclasts (14, 15). Despite this, little is known about their role in bone metastasis. In bone metastatic multiple myeloma patients, increased osteocyte apoptosis and circulating levels of pro-resorption osteocyte-specific proteins were found to correlate with increased osteolytic lesions, osteoclast formation, and bone loss (16). In preclinical models, physical connections between multiple myeloma cells and osteocytes occurred through dendrites, extensive processes used to connect osteocytes to themselves and other cells, and these connections facilitated osteolytic lesions and tumor cell growth (17). In the context of breast cancer, osteocytes secreted ATP through their dendrites in response to applied fluid flow, which inhibited breast cancer migration, proliferation, and metastasis to bone (18, 19). Based on these collective data, we speculate that osteocytes are the cellular link between mechanical loading and its inhibitory effects on bone metastatic cancer. However, whether osteocytes' mechanosensing ability is affected by bone metastasis is unknown.

We hypothesized that (i) soluble factors secreted by breast cancer cells alter the mechanoresponse of osteocytes to mechanical loading and (ii) mechanical loading of breast cancer cells modulates their effect on osteocyte mechanosensing. To this end, we first characterized the response of osteocytes to mechanical loading and to culture with tumor-conditioned media to determine the isolated effects of each of these two tumor microenvironmental factors. As dendrites are the most mechanosensitive part of the osteocyte (20, 21), and they increase in length (22) and number (23) in response to anabolic loading, particularly fluid flow (24), they are useful indicators of overall osteocyte mechanosensitivity. Next, to determine whether tumor-derived soluble factors modulated the osteocyte dendritic response to loading, osteocytes underwent mechanical loading in the presence of tumor-conditioned media. Finally, to model the more physiological situation in which both cell populations undergo loading, we exposed osteocytes to loading as well as media conditioned by breast cancer cells that had been similarly loaded. We evaluated dendrite formation and gene expression of E11/gp38, a marker of early osteocyte maturation that regulates dendrite formation during osteocyte differentiation (25). We also evaluated indicators that would point to changes to downstream remodeling, including osteocyte apoptosis, a trigger for downstream resorption (26), and gene expression of NF-κB ligand (RANKL) and its endogenous soluble inhibitor osteoprotegerin (OPG), the ratio of which is the critical factor controlling osteoclast differentiation and function (27).

## Materials and methods

### Cell culture

MLO-Y4 cells (MLOs), a gift from Dr. Lynda Bonewald, were selected as our osteocyte model when investigating the role of mechanical loading because their mechano-response to applied fluid shear stress is very robust and well-characterized (22, 28–31). MLOs were cultured on collagen-coated tissue culture plastic [rat-tail collagen I (Fisher #CB-40236) diluted to 0.15 mg/ml in sterile 0.02 N acetic acid]. MLOs were maintained in Minimum Essential Medium Eagle—Alpha Modification (αMEM) supplemented with 2.5% fetal bovine serum (FBS), 1% Penicillin/Streptomycin (P/S) and 2.5% calf serum (CS) under standard cell culture conditions (37°C, 5% CO_2_) (32). After cells reached ~70% confluency, they were subsequently seeded onto collagen-coated coverslips (2,000 cells/cm^2^) for experimentation.

### Mechanical loading

Osteocytes are particularly sensitive to fluid flow-induced shear stresses (24, 33), which changed expression of osteocyte-generated signals in favor of net bone formation, such as RANKL (34), OPG (35), and sclerostin (36). MLOs were exposed to oscillatory fluid shear stresses via a rocking see-saw platform, a simple, high-throughput system in which many standard culture dishes can be placed on a platform that rocks up and down in the vertical plane (37). This type of applied fluid flow set-up has elicited a strong anabolic response in osteocytes, including MLOs (29, 38, 39). The rocking parameters were adjusted to achieve a maximum characteristic shear stress of ~1.1 Pa [calculated using equations from Zhou et. al. (40)], a stress level shown to increase dendrite formation and length in MLOs (22) and, when applied using steady flow, alter breast cancer migration dynamics in models of primary tumors (41, 42). Rocking was performed for 2 h per day at ~1 Hz in the incubator for 3 days, after which cells were harvested for analysis.

### Generation of tumor-conditioned media

MDA-MB231 human breast cancer cells (MDA, ATCC) were maintained in complete Dulbecco's modified Eagle medium [DMEM (Invitrogen) supplemented with 10% FBS (Life Technologies) and 1% P/S (Invitrogen)] under standard cell culture conditions (37°C, 5% CO_2_). To generate tumor conditioned media (TCM), MDAs were plated in T150 flasks and when they reached 90% confluency, their media was replaced with low serum DMEM (1% FBS, 1% P/S) for 24 h. TCM was collected, concentrated 10-fold in an Amicon centrifugal filter unit (MWCO 3kDa, EMD Millipore), and diluted to 2-fold final concentration in fresh MLO media, a level that previously affected other bone cells, including osteogenic differentiation in mesenchymal stem cells (13) and osteoclastogenesis in RAW 264.7 monocytes (43). Low serum DMEM with no cells was subjected to the same processing conditions and used for control. To generate loaded conditioned media, MDA cells were plated in T150 flasks, which were randomized into Loaded and Nonloaded TCM groups. Loaded TCM flasks received rocking for 6 h to match total loading exposure in MLOs, after which media was replaced with low serum DMEM and processed as previously described.

### Fluorescent staining

To quantify MLO dendrite number and cell number, we utilized phalloidin staining (Invitrogen, A34055) and DAPI (Sigma-Aldrich, D9542), respectively. To explore whether apoptosis contributed to loading-induced changes to the dendrite/osteocyte ratio, we utilized terminal deoxynucleotidyl transferase dUTP Nick-End Labeling (TUNEL) staining (DeadEnd Fluorometric TUNEL System, Promega, G3250) to assess MLO apoptosis. Briefly, for dendrite imaging, cells were fixed using 4% paraformaldehyde, washed with PBS, and permeabilized with 0.05% Triton-X. Cells were incubated with 1:5000 of DAPI and 1:200 of phalloidin for 1 h. For TUNEL staining, cells were incubated with 1:5000 of DAPI and 1:200 of phalloidin combined with recombinant terminal deoxynucleotidyl transferase enzyme per manufacturer's instructions. Cells were then imaged using spinning disk confocal microscopy (Zeiss Spinning Disk Cell Observer SD, Carl Zeiss). Seven fields of view were randomly selected for each coverslip, from which cell number, dendrite number, and TUNEL-positive cells were quantified using ImageJ (NIH). Data from all fields of view per sample were averaged for reporting.

### Gene expression

To explore additional potential mechanisms underlying loading-induced changes to physical dendrite formation, gene expression of E11/gp38, RANKL, and OPG were determined using quantitative real-time polymerase chain reaction (qPCR) and the comparative ΔCT method (44). Briefly, mRNA was isolated using the TRIzol extraction method in RNase-free conditions. RNA purity and quantity were tested using a spectrophotometer (NanoDrop 1000; Thermo Scientific). qPCR was performed using 25–50 ng of cDNA in a final volume of 20 μL containing 2X QuantiNova Probe PCR Master Mix (QuantiNova Probe PCR Kit, 208256, Qiagen). Predesigned qPCR probe assays were purchased for each of the genes of interest (E11, Mm.PT.58.42823717, Integrated DNA Technologies) (RANKL, Mm00441906_m1, ThermoFisher Scientific) (OPG, Mm00435454_m1, ThermoFisher Scientific). Results were normalized to the reference gene TATA-binding protein (TBP, Mm.PT.58.10867035, Integrated DNA Technologies) and presented as fold change.

### Statistical analysis

The effects of treating MLOs with mechanical loading (NonLoaded MLOs versus Loaded MLOs), tumor-conditioned media (TCM versus Control), and whether TCM was also loaded (NonLoaded TCM versus Loaded TCM) were all assessed using full factorial ANOVAs (JMP v8.0, SAS Institute Inc.). When the interaction factor was significant, a *post-hoc* Tukey-Kramer means comparisons test with a Bonferroni correction was conducted; otherwise, experimental groups were pooled for analysis as appropriate to evaluate main effects. Statistical significance was set at *p* < 0.05. All experiments were replicated 2–4 times. All data is represented as mean + standard deviation.

## Results

### Mechanical loading, but not breast cancer-conditioned media, increased osteocyte dendrite formation

**Figure 1:**
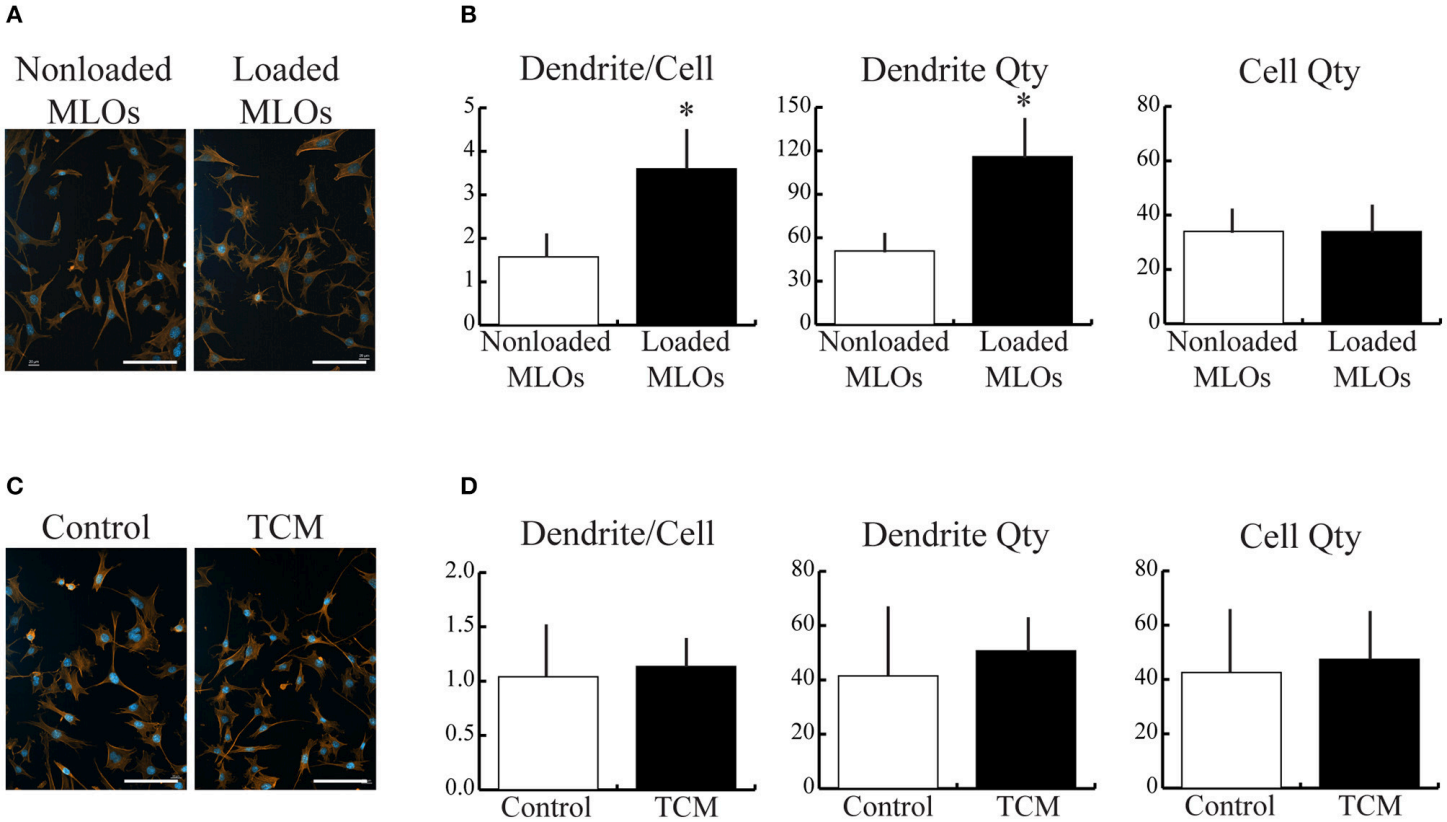
Mechanical loading, but not breast cancer cell-derived soluble factors, increased MLO-Y4 dendrite formation**. (A,C)** Fluorescent images of MLOs suggested that fluid shear stress alone increased dendrite formation, but treatment with tumor-conditioned media (TCM) alone did not. (Scale bars = 100 μm; blue = DAPI, red = phalloidin) **(B)** Image analysis revealed that mechanical loading increased the number of dendrites per MLO, which was achieved via a similar increase in overall dendrite quantity. MLO cell number was not affected by loading. **(D)** In contrast, no detectable changes in dendrites per MLO, dendrite number, or MLO cell number were observed due to TCM treatment alone. Data are represented as mean + SD. ^*^p < 0.05 relative to respective NonLoaded MLO group.

We first investigated the isolated effects of applied fluid flow and of tumor-conditioned media (TCM) treatment on MLO dendrite formation to provide a baseline response to these microenvironmental factors in our rocking system. Mechanical loading alone increased the number of dendrites per MLO primarily by boosting overall dendrite quantity (Figures 1A,B). MLO cell number was unaffected by applied fluid flow. In contrast, upon exposure to TCM, no change in dendrite/cell, cell number, or dendrite number was observed (Figures 1C,D).

### Breast cancer-conditioned media increased dendrite formation in osteocytes via a decrease in cell growth

**Figure 2:**
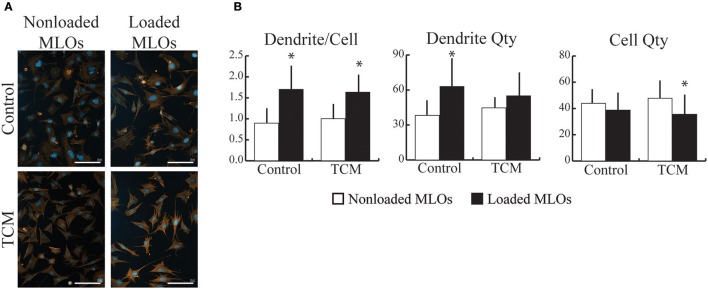
Breast cancer-derived factors modulated MLO-Y4 cell growth during mechanical loading. **(A)** Fluorescent images of MLOs revealed that mechanical loading promoted dendrite formation independent of the presence of tumor-conditioned media (TCM). (Scale bars = 100 μm; blue = DAPI, red = phalloidin) **(B)** Mechanical loading increased the number of dendrites per MLO similarly in the absence (upper) and presence (lower) of TCM. In the Control group, loading-induced changes in dendrite/cell occurred due to an increase in overall dendrite quantity whereas in the TCM treatment group, changes occurred due to a decrease in MLO growth with loading. Data are represented as mean + SD. *p < 0.05 relative to respective NonLoaded MLO group.

To determine how breast cancer cell-derived factors affected osteocytes' response to mechanical loading, we exposed MLOs to fluid shear stress in the presence and absence of TCM. In the TCM and Control treatment groups, loading increased the number of dendrites per osteocyte independent of TCM status (Figures 2A,B). Specifically, in the Control group, the loading-induced increase in dendrite/cell was driven by a rise in total number of dendrites and no loading-induced changes in MLO cell number (Figure 2B). In contrast, in the TCM group, the increase in dendrite/cell resulted from a reduction in MLO cell number and no loading-induced changes in total number of dendrites.

### Mechanical loading applied to breast cancer cells modulated their effects on loading-induced osteocyte dendrite formation

**Figure 3:**
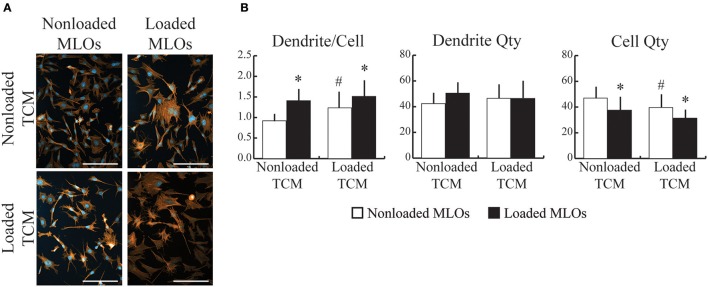
Mechanically-loaded breast cancer cells altered loading-induced MLO-Y4 dendrite formation. **(A)** Fluorescent images of MLO cultured with conditioned media collected from NonLoaded MDAs (NonLoaded TCM, upper) and loaded MDAs (Loaded TCM, lower) suggested that mechanically-loaded tumor cells stimulated MLO to form more dendrites. (Scale bars = 100 μm; blue = DAPI, red = phalloidin) **(B)** Loading increased the number of dendrites per MLO for both N NonLoaded L and Loaded TCM groups. Furthermore, in the N NonLoaded L MLO groups, dendrite per cell was greater when MLOs were treated with Loaded TCM, suggesting that loading both cell types synergistically increased the number of dendrites per MLO. Loading-induced changes to dendrite per cell was achieved primarily via reductions in cell number irrespective of whether TCM came from Loaded or NonLoaded MDAs. Additionally, in NonLoaded MLO groups, MLO quantity was further reduced when treated with Loaded TCM relative to NonLoaded TCM, suggesting that loading applied to both cell types maximizes changes to cell quantity. Data are represented as mean + SD. ^*^p < 0.05 relative to respective NonLoaded (NL) MLO control group, ^#^*p* < 0.05 relative to NonLoaded MLOs treated with NonLoaded TCM.

*In vivo*, both bone cells and cancer cells are exposed to the prevailing mechanical environment in the skeleton. To recapitulate this, we collected conditioned media from breast cancer cells that had been exposed to fluid flow, and then supplied it to MLOs during applied fluid flow. Loading increased dendrite number per MLO for both Loaded and NonLoaded TCM groups (Figures 3A,B), and this response was greatest with conditioned media from mechanically loaded MDAs, suggesting that loading applied to both MLOs and MDAs synergistically increases the number of dendrites formed in MLOs. Loading-induced increases to dendrite per cell occurred primarily through reduced MLO cell number (Figure 3B) as previously observed (Figure 2B), and these decreases in MLO cell number were maximal in the Loaded TCM group.

### Applied mechanical loading breast cancer cells increased the RANKL/OPG ratio in osteocytes

**Figure 4:**
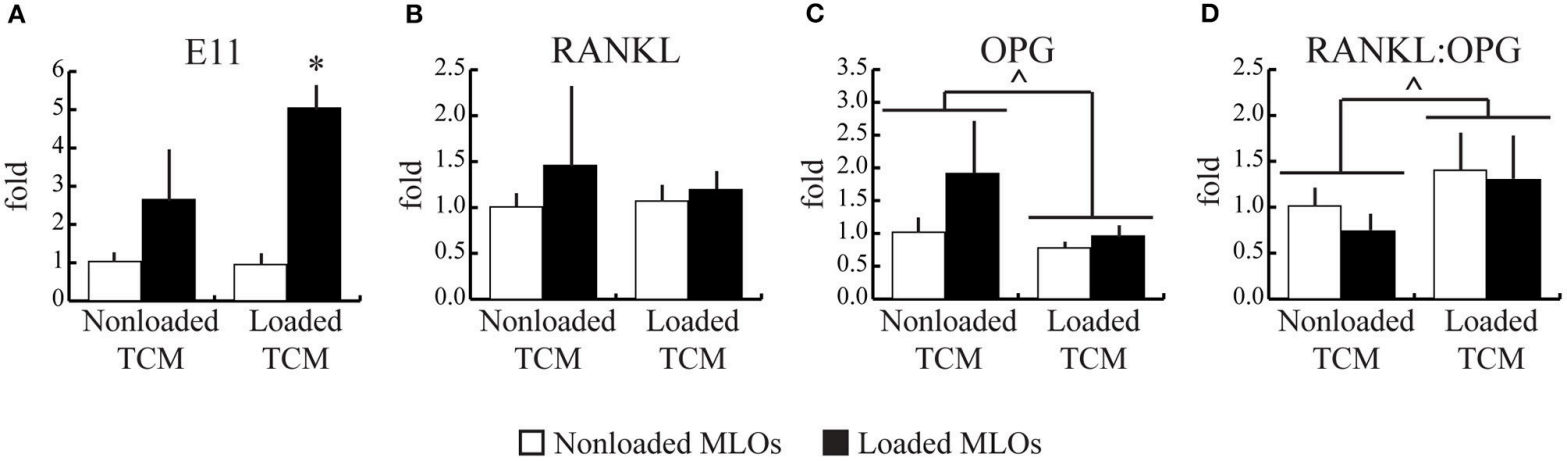
Mechanically-loaded breast cancer cells enhanced E11 gene expression and increased the RANKL/OPG ratio in MLO-Y4s. **(A)** Mechanical loading in MLOs increased their expression of E11, which regulates dendrite formation, which parallels the increased dendrite formation observed histologically and suggests heightened MLO mechanosensivity. (a trend for changes with loading is noted for the NonLoaded TCM group). **(B–D)** RANKL gene expression was unaffected by loading in both NonLoaded TCM and Loaded TCM groups while OPG expression overall was lower in the Loaded TCM group relative to the NonLoaded TCM group (main effect of TCM treatment; NonLoaded and Loaded MLOs pooled for each TCM treatment), resulting in an overall higher RANKL:OPG ratio in the Loaded TCM group. This results suggests that downstream resorption is heightened when breast cancer cells experience mechanical loading in the skeleton. Data are represented as mean + SD. ^*^p < 0.05 relative to respective NonLoaded MLO control group, ^∧^*p* < 0.05 relative to the pooled NonLoaded TCM group (main effect of TCM treatment).

To explore potential mechanisms underlying loading-induced changes to physical dendrite formation, we next quantified loading-induced changes in expression of E11, a gene related to osteocyte differentiation and dendrite formation. To further investigate osteocyte mechanosensitivity, we also quantified key genes that regulate downstream bone remodeling (RANKL, OPG). Corresponding to observed increases in dendrites per MLO, expression of E11 increased with applied fluid shear stress only in the Loaded TCM group, though a trend for loading-induced increases in the NonLoaded TCM group was present (Figure 4A). This result also suggests increased differentiation in MLOs with applied loading. No changes in RANKL gene expression occurred in response to any treatment (Figure 4B). However, treatment with Loaded TCM, though not applied loading, decreased OPG gene expression in MLOs (main effect of TCM treatment: pooled NonLoaded TCM vs. pooled Loaded TCM) (Figure 4C). This resulted in an overall increase in the RANKL/OPG ratio in MLOs (Figure 4D). Additionally, because we suspected the observed decreases in MLO cell number with TCM treatment could be due to apoptosis (17, 45), a known stimulant of resorption (26), we assessed osteocyte apoptosis via TUNEL staining, though we did not detect MLO apoptosis in any treatment group (Supplementary Figure 1).

## Discussion

To determine whether soluble signaling factors from breast cancer cells modulated osteocyte mechanosensing, we subjected osteocytes to applied fluid flow in combination with media conditioned by breast cancer cells that also underwent applied fluid flow. We found that breast cancer-derived soluble factors modulated fluid flow-induced dendrite formation in osteocytes, though they did not affect osteocytes in the absence of mechanical loading. Furthermore, when breast cancer cells underwent similar mechanical loading, their secretions impacted MLO cell growth and loading enhanced dendrite formation in the remaining osteocyte population. Soluble factors from mechanically-loading breast cancer cells increased the RANKL/OPG ratio and may also be stimulating osteocyte differentiation, as indicated by increased E11 expression. However, they do not stimulate resorption by inducing osteocyte apoptosis.

Based on our results, breast cancer cell-derived factors alone did not alter dendrite formation in MLO-Y4 cells. In a mouse model of bone metastatic multiple myeloma (MM), osteocytes and tumor cells physically interacted via dendrites, and this physical interaction was required for tumor cells to induce osteocyte apoptosis and increase levels of pro-resorption proteins RANKL and sclerostin (17). Here, we focused on paracrine signaling between breast cancer cells and osteocytes as the majority of osteocytes are deeply embedded in the bone matrix (46), although lack of physical contact could account for no observed changes to dendrite formation due to soluble factors alone and should be investigated in the future. In the context of breast cancer, when MDA-MB231 cells were treated with conditioned media collected from MLO-Y4 cells exposed to fluid shear stress (at a similar shear stress utilized in our study), the signaling molecular ATP that was secreted from dendritic hemichannels inhibited tumor cell migration, invasion, and growth (19). However, the reciprocal effects of tumor cells on osteocyte function was not investigated in that study.

When mechanical loading was applied to osteocytes in conjunction to treatment with conditioned media, loading-induced changes in dendrite formation were altered. In the control group, total dendrite number increased with loading, as expected (23). As dendrites are the most mechanoresponsive part of the osteocyte (relative to the cell body) (20, 21), increased dendrite formation is expected to confer increased osteocyte mechanosensitivity overall. Similarly, as mechanical loading is well-documented to result in net bone formation via osteocyte signaling (10), we further expect that the observed increased dendrite formation would result in downstream net bone formation, which should be verified in future studies. Our results also suggest that osteocytes are able to respond to mechanical signals despite the presence of tumor-derived factors. Similarly, when mechanical loading was applied to a mouse model of breast cancer bone metastasis, significant increases in bone mass still occurred (11). Interestingly, increases in dendrite number per osteocyte occurred in a reduced osteocyte cell population whether tumor cell-secreted factors were collected from loaded or nonloaded breast cancer cells, and reductions were maximal with conditioned media from mechanically-loaded breast cancer cells. When bone marrow-derived mesenchymal stem cells (which are osteocyte progenitors) simultaneously underwent mechanical compression and exposure to conditioned media collected from loaded breast cancer cells, their growth profile did not change, though their osteogenic differentiation did (13). Similarly, we saw evidence for enhanced osteocyte differentiation via E11 gene expression. This may indicate that effects on cell growth depend on the stage of differentiation, at least in the osteoblastic lineage. Future work is needed to identify which tumor-secreted factors are modulating osteocyte cell growth. Taken together, our data show that despite tumor-mediated reduction in osteocyte cell number, dendrite formation due to loading increased in the existing population of osteocytes, indicative of heightened mechanosensitivity.

Changes in OPG, but not RANKL, were observed in response to mechanically-loading breast cancer cell secretions and applied fluid flow. The ratio of these two factors control downstream remodeling, rather than changes in one or the other (27). Based on gene expression, we observed an overall higher RANKL/OPG ratio when loading was applied to both tumor cells and osteocytes. This is expected to correlate with either the initiation of a remodeling cycle (as osteoclast formation and resorption occurs prior to formation) or result in net-resorption bone remodeling. Moreover, previous work applying fluid shear stress to MLOs demonstrated that changes to RANK/OPG at the gene level did not correlate with changes at the protein level (35). Two hours of fluid flow at similar levels of shear stress immediately increased RANKL and OPG gene expression in MLO-Y4 cells, but RANKL protein decreased while OPG protein increased. These changes to gene expression were detected immediately following a single bout of loading, but they returned to normal within 24 h. Here, we subjected osteocytes to mechanical loading for 3 consecutive days. In the future, how changes to osteocyte gene expression correlate to changes in downstream remodeling should be investigated.

We also determined that breast cancer-mediated changes in osteocyte cell growth was not due to osteocyte apoptosis. In contrast, bone metastatic MM has been associated with increased osteocyte apoptosis (17, 45), but this effect may require cell-cell contact as mentioned previously. It is possible that reducing osteocyte growth may constitute a mechanism by which breast cancer cells avoid osteocyte-mediated inhibition of their migration and proliferation, and that mechanical loading may augment the “intrinsic self-defense mechanism” of osteocytes against this (19).

In our current work, we utilized the MLO-Y4 osteocyte cell line to perform initial experiments investigating the role of loading on modulating breast cancer-mediated changes to osteocyte function because of its well-documented response to mechanical loading, especially to fluid flow (22, 28–31). However, it is likely that osteocyte interactions with cancer cells differ depending on their stage of differentiation. For example, expression of osteocytic proteins that are associated with multiple myeloma bone metastasis, such as sclerostin, are associated with mature osteocytes (47). Here, MLO-Y4s model early osteocytes and do not secrete such proteins (32). Future work should include osteocytes at varying stages of differentiation. For example, osteocyte cell lines such as IDG-SW3s (22, 28–31) and OCY454s (48) represent mature osteocytes that secrete sclerostin, while MLO-A5s represent mineralizing osteoid-osteocytes (49), all of which are useful models to utilize in future studies. Additionally, we combined human breast cancer cells with mouse osteocyte cells. However, both of these cells lines are extensively used and studied. In particular, the MDA-MB231 cell line is widely used in mouse models of breast cancer and bone metastasis. Previously, such a mouse model of bone metastatic breast cancer was combined with mechanical loading and demonstrated that increased mechanical loading interfered with the establishment of secondary tumors and bone osteolysis (11). *In vitro* models, such as the one utilized here, can be used to help elucidate the underlying cellular mechanisms of mechanical loading on bone metastasis.

Our current experiments with MLO-Y4s were performed using a 2D system. Even though osteocytes naturally reside in a 3D lacunar-canalicular network, our approach permitted comparison of our results with that from other MLO-Y4 flow studies, which are most commonly conducted in 2D. Furthermore, by applying fluid flow in the 2D rocking set-up, the resulting shear stress profile was more controllable and better characterized in terms of estimating the shear stresses applied to the cells (whereas comparing across 3D systems, which requires computational modeling to characterize the flow field, is usually unfeasible). Thus, we were able to reliably apply a shear stress (~1 Pa) known to affect osteocyte mechano-sensing ability (22) and breast cancer cell migration (under steady flow) (41, 42). To better represent the physiological environment of osteocytes, however, future studies should be conducted in 3D systems.

In summary, we have shown that mechanically-loaded breast cancer cells modify osteocyte mechanosensing and bone remodeling by increasing dendrite formation and inhibiting OPG expression. These results highlight that osteocytes serve as the cellular link between mechanical loading and breast cancer-induced bone disease.

## Author contributions

All contributing authors have agreed to submission of this manuscript for publication. ML conceived of the study. WW, BS, GK, and ML designed experiments, analyzed data, and interpreted results. WW, BS, and GK performed experiments. WW, BS, and ML wrote the manuscript.

### Conflict of interest statement

The authors declare that the research was conducted in the absence of any commercial or financial relationships that could be construed as a potential conflict of interest.

## Supplementary material

Supplementary Figure 1 Breast cancer-derived factors did not induce MLO-Y4 apoptosis. No evidence of osteocyte apoptosis was detected due to mechanical loading or treatment with conditioned media from mechanically-loaded breast cancer cells, as assessed by fluorometric TUNEL staining. (Scale bars = 100 μm; green = TUNEL, blue = DAPI, red = phalloidin).
