# Supplementary figures and images for: Mechanically-Loaded Breast Cancer Cells Modify Osteocyte Mechanosensitivity by Secreting Factors That Increase Osteocyte Dendrite Formation and Downstream Resorption
Source: Front Endocrinol (Lausanne). 2018 Jul 3;9:352. doi: 10.3389/fendo.2018.00352 (PMC6043807; doi:10.3389/fendo.2018.00352)

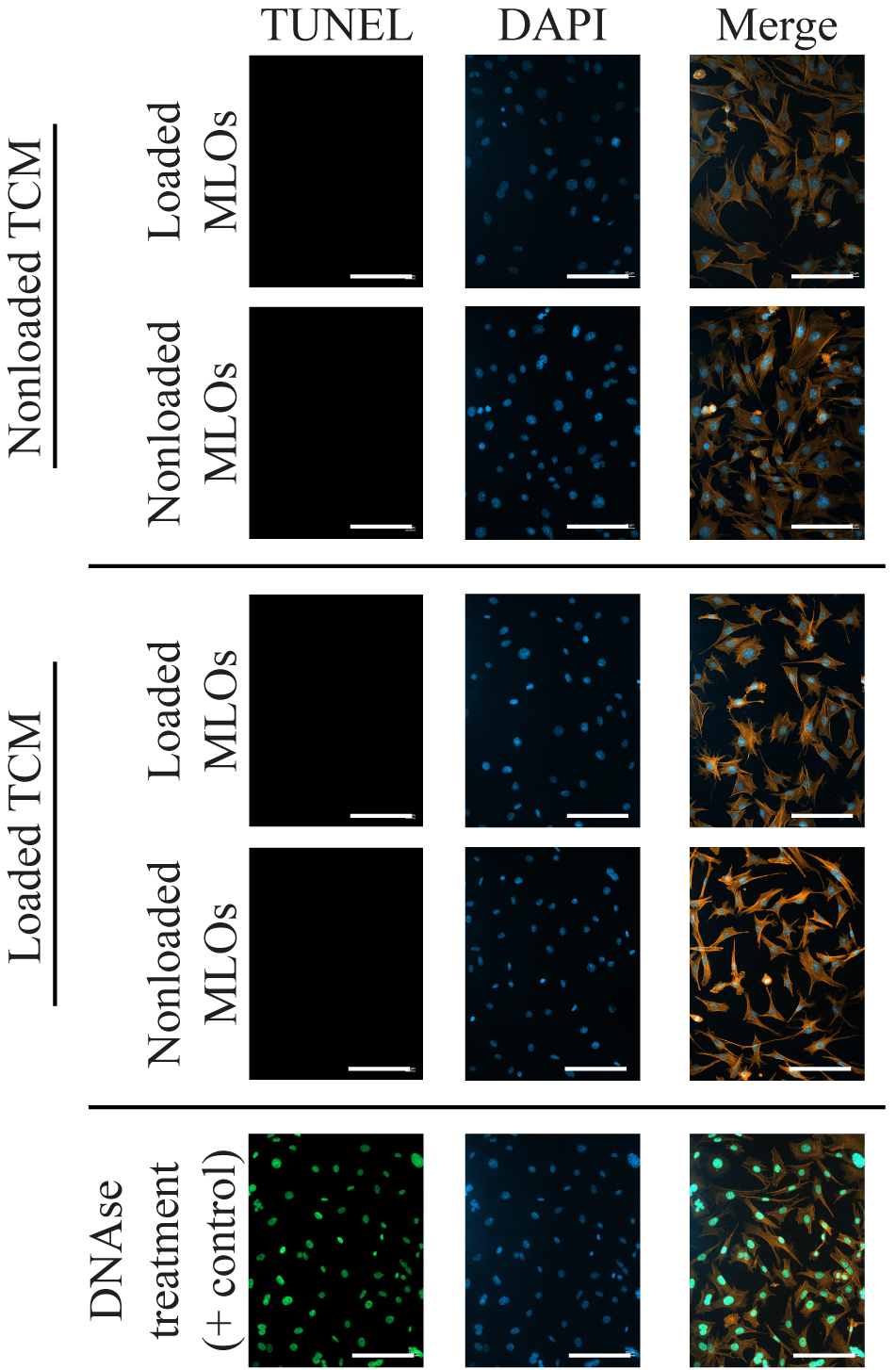

Supplement: Supplementary Figure 1 — Breast cancer-derived factors did not induce MLO-Y4 apoptosis. No evidence of osteocyte apoptosis was detected due to mechanical loading or treatment with conditioned media from mechanically-loaded breast cancer cells, as assessed by fluorometric TUNEL staining. (Scale bars = 100 μm; green = TUNEL, blue = DAPI, red = phalloidin). [file Image_1.TIF]
